# Diagnosis and Management of Pseudoguttata: A Literature Review

**Published:** 2019

**Authors:** Majid Moshirfar, Harry Y. Liu, Uma Vaidyanathan, Anisha N. Somani, Grant C. Hopping, James R. Barnes, Madeline B. Heiland, David B. Rosen, Mahsaw N. Motlagh, Phillip C. Hoopes

**Affiliations:** 1 John A. Moran Eye Center, Department of Ophthalmology and Visual Sciences, School of Medicine, University of Utah, Salt Lake City, UT, USA; 2 Utah Lions Eye Bank, Murray, UT, USA; 3 Hoopes Durrie Rivera Research Center, Hoopes Vision, Draper, UT, USA; 4 Health Science Center, McGovern Medical School, University of Texas, Houston, TX, USA; 5 School of Medicine, Virginia Commonwealth University, Richmond, VA, USA; 6 College of Medicine-Phoenix, University of Arizona, Phoenix, AZ, USA; 7 College of Medicine-Tucson, University of Arizona, Tucson, AZ, USA

**Keywords:** Pseudoguttata, Pseudoguttae, Guttae, Guttata, Endotheliitis, Secondary Guttae, Secondary Guttata, Endothelial Cell Edema, Endothelial Bleb, Endothelial Denudation, Endothelial Dystrophy, Endothelial Degeneration, Fuchs' Endothelial Dystrophy

## Abstract

Corneal pseudoguttata (PG), also known as pseudoguttae or secondary guttata, is a transient, reversible endothelial edema commonly associated with anterior segment pathology. While considered rare, PG presents on slit-lamp examination more commonly than originally thought. We have clinically observed PG after refractive surgeries, in association with infectious keratitis, and following medication use. PG presents as dark lesions on slit-lamp exam with specular illumination, similar to primary corneal guttata. PG is distinct from guttata because PG resolves over time and does not involve Descemet’s membrane. Other ocular findings that may be confused with guttata include endothelial blebs (EB) and endothelial denudation (ED). EB are possibly a type of PG that present after contact lens use or hypoxia. ED is a distinct entity that is characterized by loss of endothelial cells without involvement of Descemet’s membrane. Confocal microscopy may be useful in differentiating these four endothelial lesions, with differences in border definition and the presence of hyperreflective areas two main distinctions. PG presents as a hyporeflective, elevated shape without clear borders on confocal microscopy. PG, EB, and ED can resolve with time without the need for surgical intervention, unlike corneal guttata. Treatment of the underlying condition will lead to resolution of both PG and EB.

## INTRODUCTION

The corneal endothelium is a mono-layer of hexagonal cells attached to Descemet’s membrane. This layer is vital for maintaining the transparency of the cornea through hydration mechanisms involving the bicarbonate-ATP dependent pump, and damage to the endothelial layer can lead to serious, irreversible changes in vision [1]. Certain disorders such as Fuchs’ corneal dystrophy can cause dysfunction or death of the endothelial cells, leading to a condition known as guttata [2]. Guttata, otherwise known as guttae or true guttata, are characterized by outpouchings of Descemet’s membrane [3, 4]. The cause of Fuchs’ dystrophy has been theorized to be due to thickening of Descemet’s membrane that causes destruction of endothelial cells [2]. In contrast, pseudoguttata (PG), also known as “pseudoguttae” or “secondary guttae,” is transient and completely reversible areas of endothelial edema without Descemet’s involvement [3, 4]. PG may present clinically similar to guttata. Similarities between PG and guttata can lead to ambiguity in clinical evaluation; however, it is important to differentiate between the two since their treatment regimens differ. The purpose of this paper is to provide an overview of PG, highlighting the differences between guttata, PG, and other PG-like ocular findings such as endothelial blebs (EB) and endothelial denudation (ED), and to describe the clinical evaluation and management of these distinct entities. We review the literature covering the clinical course, differential diagnosis, etiology, pathology, treatment, and diseases associated with PG.

## METHODS

A literature search on PG was performed using the following sources: Pubmed, Google Scholar, Embase, and Scopus with keywords “guttata,” “guttae,” “pseudoguttata,” “pseudoguttae,” “pseudo guttae,” “Hassall-Henle bodies,” “guttaless Fuchs’ Dystrophy,” “Fuchs’ Dystrophy without guttata,” “endothelial bleb,” “endothelial denudation,” and “secondary guttata.” Multiple ophthalmology textbooks were examined, looking for those same keywords. There were no language restrictions. Publications were drawn between the dates of 1900-2019. Of the 139 articles found on PG, only 25 articles mentioned the ocular finding and were accessible online. Of these, 4 were case reports, 0 were review articles, and 6 articles overlapped with descriptions of the same information. A total of 19 articles provided unique information, and of these, only 14 mentioned the word “pseudoguttata” or other derivations of the word, with the other five articles reporting EB. Of the 14 articles mentioning “pseudoguttata,” eight articles examined PG in association with different conditions, and two articles examined the correlation between PG and ocular biometric data such as endothelial cell count and intraocular pressure (IOP). The majority of articles were case reports focusing on the characteristics of PG. In the publications found in this literature review, there were collectively 322 documented cases of PG.

Medical records from Dr. Moshirfar’s clinical practices at the University of Utah and Hoopes Vision from 1996-2019 were examined to find ophthalmic surgical procedures, diseases, medications, or other conditions that were noted with slit-lamp findings of PG, transient guttata, or secondary guttata. These charts were analyzed to classify associations with PG based on disease, surgery, and medication toxicity.

**Terminology**

**Figure 1:**
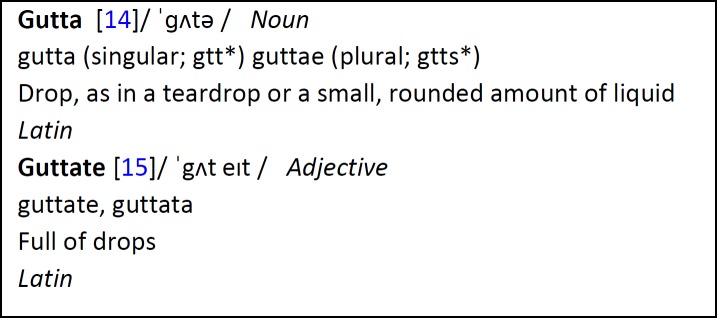
Distinctions of the Various Forms of the Word “gutta.” These Rules also apply to Words such as “pseudogutta” and “secondary gutta.” *Abbreviations for gutta commonly seen to Denote Eyedrops

Corneal guttata was first mentioned in 1921 by Vogt with the word “guttata” when describing the appearance of Fuchs’ dystrophy under a slit lamp [3, 5]. “Guttata” is an adjective that means full of drops [6]. In recent years, the Latin word “gutta,” a singular noun for the word teardrop, has been replacing the word “guttata” in the literature [6, 7]. Although neither word is incorrect, in clinical practice, “guttae” is used to describe the physical findings while “guttata” refers to the condition of having guttae [6, 7]. Figure 1 highlights these differences. Before 1921, the term was used throughout literature to describe drop-like appearances on the body, including a type of skin lesion seen in scleroderma [8]. PG, as so named due to its resemblance to guttata, was first mentioned in 1959 by Wolter and Larson to describe outgrowths of Descemet’s membrane in a patient with interstitial keratitis under the name “secondary guttae” [3, 9]. It is now understood that PG is not associated with Descemet’s membrane. Past articles dating back to 1977 have also referred to a PG-like condition using the words “endothelial blebs,” [10] which were originally thought to be associated with the same causes as PG [11-13]. From a semantic point of view, the correct term to describe the raised lesions on slit-lamp exam is “pseudoguttae.” However, many people use the word interchangeably with “pseudoguttata,” which refers to the condition or state of having endothelial cellular edema. Although we use the term “pseudoguttata” in this paper, we hope to clarify this for our readers.

It is important to define terminology before proceeding. PG is a condition involving reversible areas of endothelial edema without Descemet’s involvement [3, 4]. Guttata is characterized by outpouchings of Descemet’s membrane. EB are possibly a type of PG that present after contact lens use with endothelial cell edema, quickly resolving after removal of the causative agent [12, 13]. Due to this similarity, this article refers to EB strictly in association with contact lens use [16]. ED is loss of endothelial cells without involvement of Descemet’s membrane [17].

**Etiology**

PG is caused by intra- and inter-cellular edema [3, 4]. Most sources report PG as a condition triggered by endothelial cell injuries, iritis, corneal inflammation, or alterations in endothelial pH, with the most common cause being inflammation-associated uveitis [3, 18, 19]. An example of corneal inflammation is endotheliitis, which manifests as corneal edema and PG [20]. Endotheliitis can be caused by viral, bacterial, or fungal agents [20]. Specifically, cytomegalovirus and herpes simplex virus keratitis have both been linked to the condition [17, 21, 22]. Hypoxia causing lactate buildup in the aqueous humor also appears to be related to PG; five hours after an ultramarathon, a patient presented with guttata that resolved 48 hours later, leading us to classify the finding as PG. The mechanism was reported as oxidative stress leading to the disruption of corneal endothelium regulation [23]. Past studies suggested that high IOP can also result in endothelial edema and PG [3], but recent findings in animal studies suggest that PG can be caused by low IOP as well [24]. Topical Ripasudil, a Rho-kinase inhibitor, has been associated with morphological changes resembling PG formation, possibly due to increased cell migration and polymegathism [25, 26]. 

From 1996-2019, we have seen PG associated with many medical conditions and surgeries in our clinic. Both excimer and femtosecond assisted procedures (i.e. Laser-assisted in-situ keratomileusis [LASIK] and photorefractive keratectomy [PRK]) and manual incision surgeries (i.e. Radial keratotomy [RK] and phacoemulsification) produce PG in the post-operative period. PG has also presented during medical treatments such as intravitreal injections, antimicrobial agents, and Mitomycin-C application. For further details about these associations, refer to Table 1. We surmise that PG appears because of transient focal or localized endothelial injury, such as what occurs after surgeries and in ocular diseases. Such injury leads to disruption of the membrane pumps in the endothelial cells; however, this disruption is not severe enough to cause permanent dysfunction.

**Epidemiology**

In the last eighty years, there has only been one published article on the incidence of PG, reported as 1.1% in one clinical ophthalmology practice. In that same practice, the mean age of patients presenting with PG was 41.1 years; however, this may be more indicative of the average age of patients who undergo ophthalmic procedures [18]. In general, it is difficult to conclude a precise measure of incidence or demographic patterns associated with PG due to its short-lived nature [3]. This is perhaps why there is a shortage of literature reporting PG incidence.

**Genetics**

PG is associated with only one specific genetic disorder: keratoendotheliitis fugax hereditaria, which is an autosomal dominant, autoinflammatory disorder of the cornea resulting in corneal opacities and unilateral attacks of pain, injection, and photophobia [27]. Recently, mutations in the leucine-rich repeat (NLR) family, pyrin domain-containing 3 (NLRP3) gene have been implicated in the cause of keratoendotheliitis fugax hereditaria in a Finnish population. This disease is unrecorded in other ethnicities; however, more genetic links to PG may be uncovered with future investigations [27]. 

**Table 1:** Common Conditions, Surgical Procedures, and Medication Toxicities presenting with Pseudoguttata in Dr. Moshirfar’s Clinical Practices at the University of Utah and Hoopes Vision

Conditions and Surgeries Associated with Pseudoguttata
Infectious keratitis/iritis/endotheliitis
**Viral (herpes simplex virus, varicella zoster virus, Epstein-Barr Virus, cytomegalovirus)**
**Bacterial**
**Fungal**
**Parasitic**
Post-surgical inflammation
**Excimer laser (photorefractive keratectomy, laser-assisted in-situ keratomileusis)**
**Femtosecond laser (laser-assisted in-situ keratomileusis, small-incision lenticule extraction)**
**Radial Keratotomy (RK)**
**Superficial Keratectomy**
**YAG Laser (iridotomy, capsulotomy)**
**Pterygium excision**
**Phacoemulsification**
**Intraocular lens (IOL) explantation/implantation**
**Ultraviolet (UV) Collagen Crosslinking**
**Glaucoma laser surgery**
**Glaucoma trabeculectomy**
**Phakic IOL implantation**
**Conductive keratoplasty**
**Laser thermal keratoplasty**
**Deep anterior lamellar keratoplasty**
**Vitreoretinal procedure with and without gas/fluid exchange**
Medication toxicity
**Fortified vancomycin**
**Benzalkonium chloride (BAK) toxicity**
**Toxic anterior segment syndrome (TASS) related injury**
**Miostat**
**Mitomycin C**
**Intravitreal injection (anti-vascular endothelial growth factor, antibiotics)**
**Anti-glaucoma medications, Rho-kinase inhibitors, angiotensin-converting-enzyme inhibitors**
**Chemical (Palytoxin)**
Other conditions
**Endophthalmitis**
**Uncontrolled open-angle glaucoma**
**Angle-closure glaucoma**
**Blunt injury: airbag, human fist, head trauma**
**Thermal and chemical injury**
**Contact lens keratopathy**
**UV and infrared (IR) injury: welding, skiing, sunbathing, sunburn**
**Lactate buildup (ultramarathon)**
**Hypoxia**

Corneal guttata, on the other hand, is associated with Fuchs’ endothelial cell dystrophy (FECD), and it is characterized mostly by autosomal dominant inheritance and mutations in the COL8A2 gene [2-4, 28, 29]. Corneal guttata can also occur in isolation in an autosomal dominant pattern, without any association to Fuchs’ dystrophy [3, 4, 28].

**Diagnosis**

**Clinical Evaluation**

It is difficult to clinically differentiate between guttata and PG as traditionally, PG is a diagnosis of exclusion. Patients with PG may present with a previously documented guttata-like appearance that resolved over time. Patients with PG are typically asymptomatic [3, 18].

**Figure 2:**
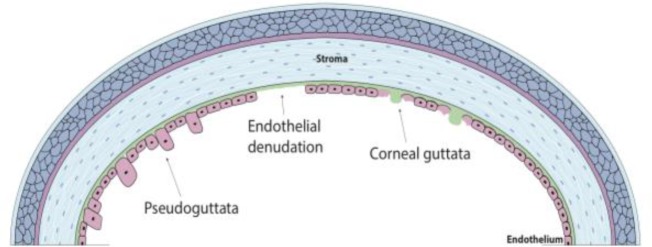
Image Showing a Cross-Section of the Corneal Layers Differentiating Endothelial Cells in Corneal Guttata, Pseudoguttata, and Endothelial Denudation. The Normal Mosaic Pattern of Endothelial Cells in Pseudoguttata Contrast with the Destruction of the Surrounding Endothelial Cells in corneal Guttata. Corneal Guttata Presents with Excrescences of Descemet’s Membrane while Descemet’s Membrane is Left Intact without any Irregularities in Endothelial Denudation and Pseudoguttata

PG appears similar to guttata on slit lamp with specular illumination, showing dark lesions and outgrowths on the corneal endothelium [3, 18]. However, endothelial cells surrounding PG are mostly unaffected, retaining a regular mosaic pattern (Fig. 2) [3, 30]. Guttata presents under the slit lamp as mushroom-like excrescences or projections of Descemet’s membrane considered to be a sign of aging or damaged endothelial cells [30]. Guttata may present with corneal edema on slit lamp secondary to endothelial cell loss in patients with Fuchs’ dystrophy [2]. Interestingly, one article mentioned a slight increase in central corneal thickness with the presence of PG, although the clinical significance of this is unknown as slit-lamp exam does not normally show corneal edema with PG [4]. On slit lamp, EB may appear similar to PG, and ED is undetectable [17].

**Histopathologic Evaluation**

Based on the current literature, histopathologic evaluation may help differentiate between PG and guttata. Histological staining with nitroblue tetrazolium stain is negative in endothelial cells with damaged nuclei, such as those seen in corneal guttata, and would exhibit an abnormal endothelial pattern [30]. The presence of PG does not impact endothelial cell density as neighboring cells are unaffected by the edema [31].

Other sources of PG, such as infection, can result in a cellular reaction and polymorphonuclear infiltration of the anterior stroma and a guttata-like appearance [3]. This infiltration has also been shown with herpes simplex virus keratitis, but it is unclear whether the inflammatory infiltrate was a result of the PG or the keratitis [17]. Confocal, specular, and light microscopy can be used to supplement history and slit-lamp exam findings of PG, guttata, EB, and ED. Confocal microscopy specifically may be used to differentiate among these four lesions. Table 2 summarizes the differences among these conditions.

**Confocal Microscopy**

Confocal microscopy shows both PG and guttata as dark, elevated shapes without sharp borders [17]. Guttata presents with a white dot in its center, unlike PG [17]. It has been theorized that the white spot occurs due to the reflection of light from the apex of the edema while the dark area results from the sides of the cell reflecting light away from the microscope [16]. EB and PG can be distinguished on confocal microscopy; EB appear as dark, elevated circles with a hyperreflective white dot in their center [16, 17]. PG, which is also characterized by cellular edema, does not show this hyperreflective spot, suggesting that more research is required to explain these findings. In contrast to the borderless PG, blebs appear sharp and well-defined [17]. ED appears as a large, sharply-defined hole [17].

**Specular Microscopy**

**Figure 3:**
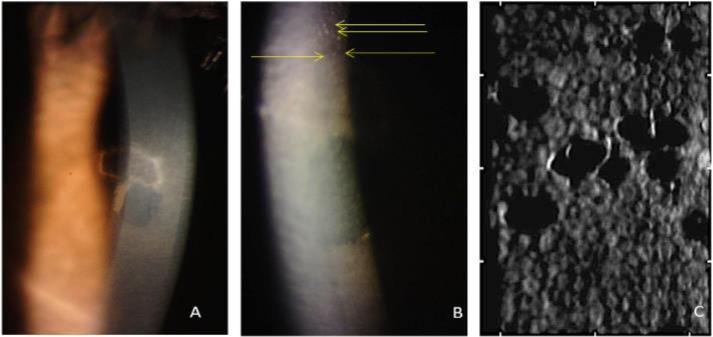
Pictures taken of a Patient who presented with Pseudoguttata following Collagen Cross-Linking for Endotheliitis. A: A Resolving Epithelial Defect after Collagen Cross-linking. B: Slit-lamp exam showing Pseudoguttata as Raised Dots. The Arrows Highlight Pseudoguttae. C: Image of Pseudoguttata taken with a Specular Microscope showing the Hexagonal Mosaic of the Endothelial Cells and the Large, Dark Regions representing these Lesions

Assessment of PG with specular microscopy reveals numerous dark holes of different sizes (Fig. 3) [3, 18]. EB and ED may appear similar to PG on specular microscopy, also presenting as dark lesions.

**Light Microscopy**

Guttata can be distinguished from PG by its characteristic outpouching appearance on light microscopy, caused by the overlying and surrounding endothelial cells that degenerate and deposit increased basement membrane [28, 30].

**Differential Diagnosis**

Guttata commonly affects middle- to older-aged patients and is seen mostly in the center of the cornea [28]. Peripheral corneal guttata, also known as Hassall-Henle bodies, presents mainly in younger patients [3, 28]. Corneal guttata may occur more frequently in women, but this association remains inconclusive in the current literature [18].

Etiology of corneal guttata must also be considered because the ambiguity of PG may lead to misreporting of clinical findings. Iridocorneal Endothelial Syndrome (ICE), Chandler’s syndrome, angle-closure glaucoma, relative anterior microphthalmos, Brown-McLean syndrome, posterior polymorphous corneal dystrophy (PPMD), and RK have been associated with corneal guttata-like changes on histopathological examination [32-38]. RK appears to be an example of a nonhereditary cause of guttata, which is characterized by endothelial degeneration [38]. 

**Table 2:** Differentiation between Gutta, Pseudoguttata, Endothelial Blebs, and Endothelial Denudation Using Confocal and Specular Microscopy

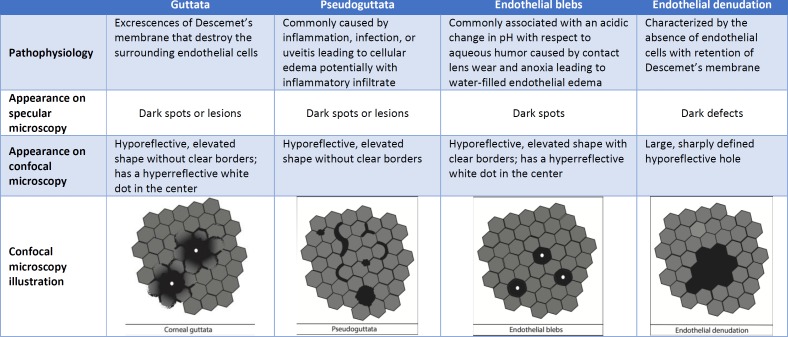

We surmise that PG, EB and ED are a spectrum of clinical manifestations of transient endothelial cell injury of various causes. EB can be seen within ten minutes of insertion of a contact lens and increase rapidly in number before partially resolving after forty-five minutes [16]. Interestingly, the density of blebs has been reported highest in patients of Asian ethnicity [16]. They appear due to a stressed corneal endothelium, and chronic conditions may lead to a decrease in endothelial cell count [13, 16]. 

EB were reported following anoxia, with the underlying cause attributed to the acidic change in pH [16, 19]. Any source of hypoxic stress, such as contact lenses, has also been associated with polymegathism [39]. The incidence of EB has been theorized to be 100% in contact-lens wearers [16]. The acidic change in pH with respect to the aqueous humor leads to water-filled vacuoles accumulating in endothelial cells, causing cellular edema. As Descemet’s membrane provides more resistance than the aqueous humor, EB project posteriorly into the anterior chamber [11, 16]. Like PG, EB are transient [17, 40]. As previously mentioned, ED is characterized by loss of endothelial cells without any insult to Descemet’s membrane. They generally take weeks to heal, and endothelial cell loss has reportedly been caused by endothelial contusion, glaucoma, and surgeries [17, 41].

**Management and Prognosis**

PG, EB, and ED do not require any surgical intervention. There are many clinical incidences where PG has presented on slit-lamp exam but resolved without any intervention the following day. However, some external causes of PG such as inflammation, uveitis, or infection will disappear within days after resolution of the underlying etiology [3, 30, 31]. For example, PG caused by inflammation may require prednisolone, while PG caused by bacterial infection may require antibiotics [3].

PG can appear in a variety of situations, without resultant harm or lasting damage [18]. 

EB typically resolve within minutes after removal of the contact lens. However, some reports have noted spontaneous resolution of blebs even with the continuous use of contact lenses [18]. ED persists for weeks after cessation of the insult but will disappear with the migration of reserve endothelial cells, similar to what occurs after Descemet’s stripping without endothelial keratoplasty (DWEK) [17, 42]. Thus, ED is the result of DWEK and can be seen in patients undergoing this treatment for early-stage Fuchs’ dystrophy. After DWEK, Rho-kinase inhibitors are often used to accelerate the rate of endothelial cell migration and can possibly treat other forms of ED [42, 43].

## CONCLUSION

Understanding PG is important in order to differentiate various ocular exam findings. In recent literature, PG has been defined as transient endothelial cell edema that appears similar to primary corneal guttata on slit-lamp examination. With the numerous conditions we have seen associated with PG, we argue that PG occurs more frequently than previously reported. EB may be a specific type of PG associated with contact lens wear. Although EB appear differently on confocal microscopy, this may be due to edema caused by water-filled vacuoles rather than inflammatory infiltrate. Their underlying causes are different, but clinicians should manage EB and PG in a similar manner. Corneal guttata and ED are defined by damage to endothelial cells. However, ED resolves with migration of endothelial cells while guttata is characterized by more lasting damage. In addition to permanent endothelial damage, guttata has excrescences of Descemet’s membrane. PG, EB, ED, and corneal guttata can be differentiated on confocal microscopy. Studies are still reporting new findings and associations with PG, and our understanding of these clinical manifestations will increase with time. Guttata is a disease process, indicative of permanent damage that may lead to vision loss. It is important to distinguish PG from guttata in order to tailor appropriate treatments. Having a better understanding of the two can assist physicians in appropriate diagnosis and clinical management.

## DISCLOSURE

Ethical issues have been completely observed by the authors. All named authors meet the International Committee of Medical Journal Editors (ICMJE) criteria for authorship of this manuscript, take responsibility for the integrity of the work as a whole, and have given final approval for the version to be published. No conflict of interest has been presented.

## Funding/Support:

Research to Prevent Blindness, NY, USA.
